# Local dermal application of a compound lidocaine cream in pain management of cancer wounds

**DOI:** 10.1590/1414-431X20198567

**Published:** 2019-11-07

**Authors:** L. Peng, H.Y. Zheng, Y. Dai

**Affiliations:** 1 Cancer Center, Union Hospital of Tongji Medical College, Huazhong University of Science and Technology, Wuhan, Hubei, China; 2 Department of Hand Surgery, Union Hospital of Tongji Medical College, Huazhong University of Science and Technology, Wuhan, Hubei, China; 3 Department of Nursing, Union Hospital of Tongji Medical College, Huazhong University of Science and Technology, Wuhan, Hubei, China

**Keywords:** Local, Compound lidocaine cream, Cancer wound, Pain

## Abstract

The aim of this study was to explore the analgesic effect of local application of compound lidocaine/prilocaine cream on cancer wounds during wound care in order to reduce the amount of morphine intake or completely replace the systemic morphine administration and optimize the protocol for cancer wound pain management. All patients were enrolled with a visual analog scale (VAS) pain score ≥4. Before wound care, 60 patients were randomly divided into 2 groups of 30 each: morphine group (10 mg tablet); topical 5% compound lidocaine cream group (0.2 g/cm^2^). VAS scores, heart rate, and Kolcaba comfort level were recorded for the two groups 10 min before and 10, 15, 20, and 25 min after wound care and data were analyzed statistically. The means for the pain score and heart rate of the topical lidocaine/prilocaine cream group were lower than those of the morphine group (P<0.01) and the Kolcaba comfort level was higher (P<0.01). Local dermal application of the compound lidocaine cream can be used as an alternative to the systemic morphine administration in cancer wound care for its safety and effectiveness. In addition, it can improve the patients' comfort and quality of life.

## Introduction

In general, 55–95% of patients with cancer wounds suffer with wound pain (1,2), which releases various stress hormones causing a severe stress response in the body that may interfere with normal physical and psychological status (3,4). Cancer wound pain is a complex pathophysiological process. Chronic wounds caused by long-term inflammatory reaction stimulate local skin afferent receptors and increase the sensitivity of peripheral nerves, leading to neuropathic pain. In addition, cancer cells compress the wound bed tissue or erode the peripheral blood vessels and nerves, resulting in severe pain.

At present, most of the compound lidocaine creams used in domestic and foreign studies are used for skin mucosa puncture or superficial preoperative infiltration anesthesia. There are some reports on the use of morphine gel in the pretreatment of pain for dressing change of cancer wounds (5), but there is no correlation study in China. Management of cancer wound pain remains a great challenge for wound care and nursing, according to current data. Therefore, it is of profound significance to select topical painkillers with quick onset, long analgesic time, and safe to relieve wound pain and improve patient comfort. Considering the effective analgesic effect of compound lidocaine cream as an infiltrating anesthetic in cortical pain receptors and nerve endings, this study explored its application in the skin of incomplete cancer wounds as a method of pain intervention.

## Material and Methods

### Subjects

From June 2015 to December 2017, 60 cases of patients with cancer wound pain treated in the wound outpatient department of the tumor center of a third-grade general hospital were selected as research subjects, including 24 cases of breast cancer, 2 cases of vulvar cancer, 8 cases of hypopharyngeal cancer, 16 cases of soft tissue sarcoma, 4 cases of laryngeal cancer, 4 cases of tongue cancer, and 2 cases of adenocarcinoma in 24 males and 36 females. The mean age was 51.4±16.26. Inclusion criteria were 1) in line with the pathological or imaging diagnosis of cancer wound; 2) pain in wound area with visual analog scale (VAS) score >4; 3) wound dressings were applied with non-viscous or low-viscous dressings; 4) signed informed consent form for voluntary participation in this study. Exclusion criteria were cancer wound with other skin complications and allergy to amides local anesthetics.

### Methods

Patients were randomly divided into two groups in chronological order, with 30 cases in each group: a 10-mg morphine hydrochloride tablet (Qinghai Pharmaceutical Factory, China) was given orally 10 min before dressing change or 5% compound lidocaine cream (containing 2.5% lidocaine/2.5% prilocaine, TongFang Pharmacy, China) was applied 10 min before dressing change, with a dose of 1.5 g/10 cm^2^. Compound lidocaine cream is a mixture of 2.5% lidocaine and 2.5 prilocaine. Before application, the secretions on the wound surface were gently washed with normal saline, aimed at maximizing wound surface absorption. Normal saline was used to wash the wound in both groups, and the secretions attached to the wound were swabbed with cotton balls and normal saline. Necrotic and exfoliated tissue was treated with conservative mechanical debridement. Bleeding was contained by applying gauze pressure. During wound management, patients were asked to rate their feeling of pain and their heart rate was monitored. After treatment, metronidazole gel (Coloplast, Denmark) was applied to the wound surface (6). Non-viscous dressing was used as the inner layer of the wound and cotton pad or foam was used for the outer layer.

Nurses involved in dressing change had at least 2 years of experience, and the operation was conducted by the same specialized nurse under the guidance of the wound doctor. The dressing change interval was no more than 2 days, and the wound treatment was completed within 30 min to ensure the consistency of the data.

The VAS pain score, heart rate, and Kolcaba comfort level of the two groups were recorded before and after the intervention. During the process of dressing change, the data was collected at 5 time-points (10 min before, and 10, 15, 20, and 25 min after medication) to observe the changes of patients' pain scores and heart rate.

### Assessment criteria

The VAS (7,8) and facial pain expression scale were used to evaluate pain. VAS scores range from 0 to 10, being 0 no pain, 1–3 mild pain (pain does not affect sleep), 4–6 moderate pain, 7–9 severe pain (inability to sleep or waking up during sleep due to pain), 10 most severe pain. It may be difficult to determine the pain level in patients who are unable to communicate using the above method. In such cases, pain level can be assessed by giving scores to different facial expressions, being 0 no pain, 1–3 mild pain, 4–6 moderate pain, 7–9 severe pain, and 10 worst pain imaginable. Heart rate was measured with a sphygmomanometer (Panasonic, Japan) at the different time-points. Comfort was measured by the Kolcaba comfort scale, which has 28 items in 5 dimensions: physiological, psychological, spiritual, socio-cultural, and environmental rated in a 1–4 Likert-format scale, with 1 indicating strongly disagree and 4, strongly agree. Negative items were rated as 1 for strongly agree and 4 for strongly disagree. The higher the score, the higher the comfort. After the procedure, each patient self-completed the table under the guidance of the staff; the response rate was 100%.

### Statistical analysis

SPSS15.0 statistical software (IBM, USA) was utilized to analyze the data. Normally distributed data were compared with the independent samples *t*-test and are reported as means±SD. Measurement data that were not consistent with normal distribution were compared with non-parametric tests and are reported as median and interquartile range. P<0.05 indicated that the difference was statistically significant.

## Results

**Figure 1.**
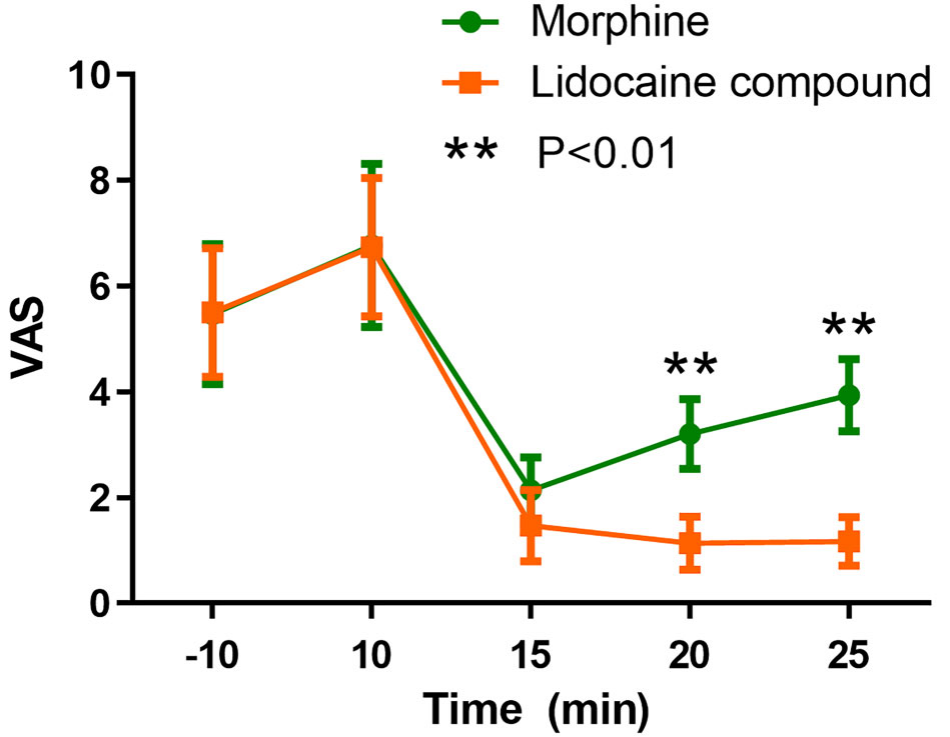
Comparison of pain levels using visual analog scale (VAS) scores between two groups of patients receiving systemic morphine or topical lidocaine for cancer wound dressing change. Data was collected 10 min before the treatment and 10, 15, 20, and 25 min after. Student's *t*-test was used for statistical analysis (P<0.01).

**Figure 2.**
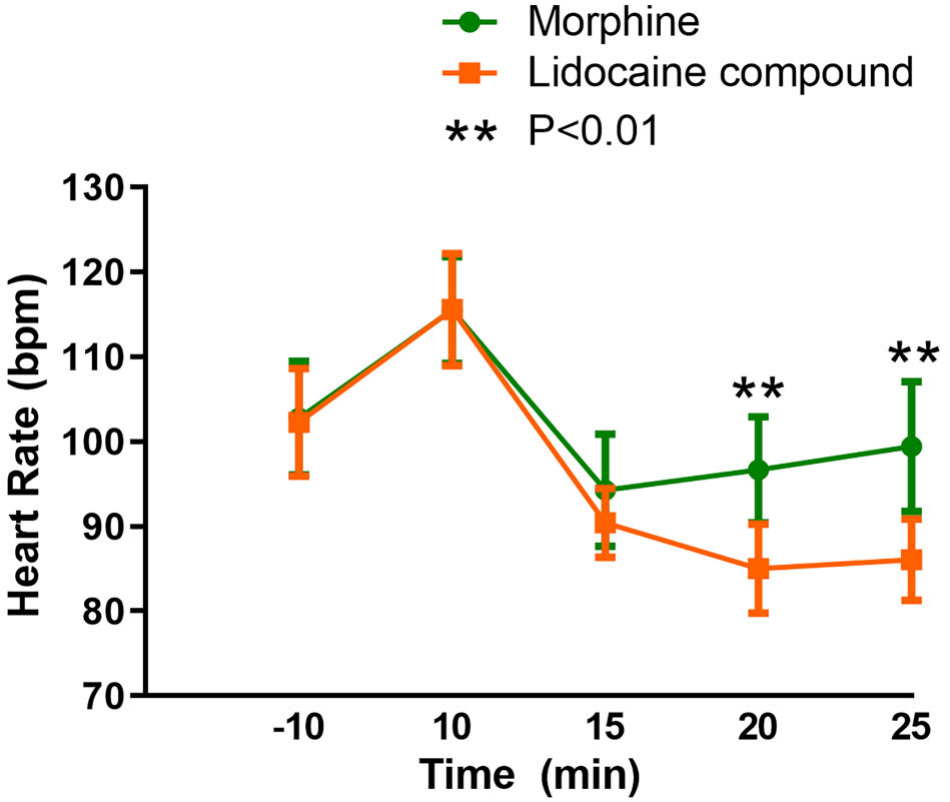
Comparison of heart rates in different time periods between the two groups receiving systemic morphine or topical lidocaine for cancer wound dressing change. Data was collected 10 min before the treatment and 10, 15, 20 and 25 min after. Data are reported as means±SD. Student's *t*-test was used for statistical analysis (P<0.01).

**Figure 3.**
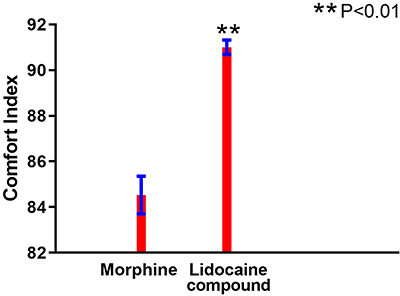
Comparison of comfort ratings between two groups of patients receiving systemic morphine or topical lidocaine compound for cancer wound dressing change. Data are reported as means±SD. Student's *t*-test was used for statistical analysis (P<0.01).

Baseline comparisons between the two groups are shown in Table 1. No statistically significant differences between the two groups were found for pain area, amount of fluid seepage, wound odor, and wound area (P>0.05). In this study, the experimental use of 5% compound lidocaine on the cancer wound surface had a significant analgesic effect. As can be seen from the curves in Figure 1 and Figure 2, both pain and heart rate of the patients in the two groups showed a peak 10 min after the drug intervention during the cleaning stage, followed by a decrease and an upward trend at the end of dressing change, but the score was still slightly lower than before the intervention. The compound lidocaine group showed the advantages of long-term analgesia 15 and 20 min after the intervention. In terms of pain relief, the lidocaine effect was faster and longer than 10 mg morphine tablets and it maintained a near painless state for a long time. Comfort ratings were also higher for the group that received topical lidocaine (Figure 3).

**Table 1.** Basic information of wounds in the two groups of patients receiving systemic morphine or topical compound lidocaine for cancer wound dressing change.

Group	Morphine	Lidocaine	Statistics	P
Wound area (n)			1.644	0.649
Neck	14	10		
Body	6	4		
Limbs	8	10		
Perineum	2	6		
Drainage quantity (n)			0.844	0.656
Large	16	14		
Medium	4	8		
Small	10	8		
Pain area (n)			0.868	0.648
Middle	6	8		
Margin	16	18		
Both	8	4		
Odor (mark/n)			1.588	0.811
5	14	10		
4	4	4		
3	6	6		
2	4	8		
1	2	2		
Area in cm^2^ (mean±SD)	54.93±76.878	51.62±76.797	1.058	0.229

Statistical analysis was done with Student’s *t*-test.

## Discussion

Compound lidocaine cream is a mixture of 2.5% lidocaine and 2.5% prilocaine, which are superficial anesthetics that can penetrate the entire skin and mucosa (9). The commonly used prilocaine is a long-term local anesthetic. The anesthetic creates an opportunity for the operator to perform debridement to control infection and reduce pain caused by inflammatory reactions, while the long-term effect reduces the need for oral opioids for wound healing pain (10).

Galluzzi et al. and Alvarez et al. believed that individualized management of cancer wound pain must be built on a comprehensive assessment of the organism (11,12). Adequate doses of analgesics can manage pain, but they can also affect psychological and cognitive function, and this side effect should be considered. In the National Comprehensive Cancer Network (NCCN) adult cancer pain guidelines 2016, 2nd edition, it is pointed out that opioids can be used for treatment operations, such as pre-analgesic treatment before wound care, as the release of morphine has a significant effect in relief of pain outbreak. The current routine dose is 10 mg/time, which is contraindicated for elderly patients and children. The use of morphine has various complications such as respiratory depression, gastrointestinal reaction, and addiction. At the same time, oral morphine sustained-release tablets are also common drugs for the treatment of moderate and severe cancer pain. However, due to side effects such as nausea, vomiting, drowsiness, and respiratory depression, patients' compliance becomes worse. In addition, sufficient time should be given between opioid administration and wound treatment to maximize the analgesic effect (13).

Due to the fragile characteristics of cancer wound tissue and a rich vascular network, the methods used in the dressing change process should be cautious, thus the dressing change time is longer than the general chronic wound dressing time. Both groups reached the peak point of the pain curve during wound cleaning, even higher than the score before intervention. This indicated that both mechanical friction and irritation of the cleaning solution during the cleaning process can result in increased pain. Therefore, drug intervention should be done earlier, and the next step of treatment should be started after the drug effect is at its peak, but this time-point still needs to be further studied. Moreover, the temperature of the cleaning solution and the method of the cleaning process could be improved to reduce pain.

Heart rate change is an objective measure of pain response. Pain stimulus can cause the release of endogenous and active substances in the patient's body, accelerate heart rate, and increase myocardial oxygen consumption. In the debridement process, the pain of patients is worse than during cleaning, due to the friction on the wound surface. This shows that friction pain is more intense than pain inflicted by the use of scissors. During the experiment, we also found that pain was not uniform in the whole wound, but more intense at the edge between the ulceration surface and normal skin, which may be related to the stimulation of local skin afferent receptors and increase in the sensitivity of peripheral nerves to pain. This is why we should treat the area at the edge of the wound as gently as possible to reduce friction and other irritants during dressing change.

Pain is a subjective feeling, and the comfort curve shows that patients who received 5% lidocaine cream had higher comfort than patients who took morphine tablets orally. In an international study (14) of 11 countries, cancer wound patients reported the most intense and unbearable pain during dressings changes (15). Inappropriate preparation for dressing change can increase patients' painful feelings dramatically, and the painful feeling can be carried over to the next dressing change step, which will increase the pain feeling. In recent years, studies show that cancer wound care is not just limited to symptom control. Different strategies should integrate palliative, integrative, and empathetic care towards patients' overall well-being. Non-drug methods to relieve pain and anxiety can help patients control pain by diverting their attention. The suggestions include relaxation, music, massage, video, meditation, and aromatherapy.

We concluded that topical compound lidocaine cream could decrease the use of systemic morphine and its side effects during wound dressing change. In addition, it was safe and provided prolonged pain relief after dressing change procedures.
